# Optimising Radiotherapy for Prostate Cancer: Comparison of Treatment Schedules and Toxicities

**DOI:** 10.7759/cureus.107249

**Published:** 2026-04-17

**Authors:** Kanumuri Asha, Lakshmi JS, Sathya Narayanan MS, Hemavathi TM, Satish Srinivas, Christopher John

**Affiliations:** 1 Radiation Oncology, Sri Ramachandra Institute of Higher Education and Research, Chennai, IND; 2 Surgical Oncology, Sri Ramachandra Institute of Higher Education and Research, Chennai, IND

**Keywords:** biochemical control, prostate cancer radiation therpay, prostate hypofractionation, radiation-induced toxicity, vmat radiotherapy, volumetric-modulated arc therapy

## Abstract

Background

Prostate cancer is one of the most common malignancies diagnosed among men worldwide, ranking second in terms of incidence, and is managed using a multimodality treatment approach. Radiotherapy remains a cornerstone in the management of localized and locally advanced disease. Although conventional fractionated radiotherapy (C-RT) has historically been the standard of care, accumulating radiobiological evidence supports the use of hypofractionated regimens. Prostate cancer exhibits a low α/β ratio (typically 1.5-3 Gy), indicating greater tumor sensitivity to larger fraction sizes and providing a strong radiobiological rationale for hypofractionated radiotherapy (hypo-RT) without escalating late toxicity to surrounding normal tissues.

Aim

This study aimed to compare biochemical recurrence-free survival (bRFS) and toxicity profiles between moderate hypo-RT and C-RT.

Materials

In our study, we analysed the data of 20 patients treated between January 2022 and January 2024. Patients received either moderate hypo-RT (70 Gy in 28 fractions) with an EQD2 of 77 Gy or C-RT (80 Gy in 40 fractions). Treatment planning involved daily cone beam CT (CBCT)-based image guidance and strict bladder-rectal protocols. Target delineation adhered to the European Society for Radiotherapy and Oncology (ESTRO)-European Organisation for Research and Treatment of Cancer (EORTC) contouring guidelines. Toxicities were assessed using the Radiation Therapy Oncology Group (RTOG) criteria and quality of life (QoL) by the International Prostate Symptom Score (IPSS).

Results

At a median follow-up of 24 months, bRFS was comparable between the two treatment groups. Grade ≥2 gastrointestinal (GI) and genitourinary (GU) toxicities did not differ significantly between hypo-RT and C-RT arms (p>0.05). Urinary symptom-related outcomes were similar across groups, with no statistically or clinically meaningful differences observed.

Conclusion

Moderate hypo-RT takes advantage of the radiobiological properties of prostate cancer with its low α/β ratio in achieving comparable oncologic outcomes with minimal early and late toxicities in intermediate-risk and high-risk prostate cancer without affecting the QoL.

## Introduction

Prostate cancer is among the common malignancies seen worldwide. According to GLOBOCAN 2020, it ranks fourth most common overall and second most common among men globally, with an age-standardized incidence rate (ASIR) of 30.7 per 100,000 men. In India, it ranks sixth among the causes of mortality in men. Median age at diagnosis is approximately 65 years. Newly detected prostate cancer accounted for an estimated 37,948 and 18,386 deaths in India, corresponding to an ASIR of 5.6 per 100,000 men and an age-standardized mortality rate of 2.7 per 100,000 [1].

Over the last decade, advances in understanding tumor radiobiology have transformed radiotherapy practice in prostate cancer, shifting from conventional fractionation schedules towards hypofractionation and stereotactic body radiotherapy approaches [2,3,4]. The key radiobiology feature that makes prostate cancer unique is its low α/β ratio (3.1 Gy), which is similar to that of late-responding normal tissues [4].

The Conventional or Hypofractionated High-dose Intensity-Modulated Radiotherapy for Prostate Cancer (CHHiP), Radiation Therapy Oncology Group (RTOG) 0415, Prostate Fractionated Irradiation Trial (PROFIT), and Hypofractionated versus Conventionally Fractionated Radiotherapy (HYPRO) trials have supported the safety of a hypofractionated schedule in the radiotherapy management for intermediate- and high-risk prostate cancer [2, 5-7]. However, there is limited data [8,9] on the clinical outcomes and tolerability of hypofractionated radiotherapy (hypo-RT) in the Indian population. The other Indian data in the aforementioned studies have used other hypofractionated schedules in contrast to the one used in our study. Hence, we conducted a retrospective study to assess efficacy and toxicity at our institution.

## Materials and methods

A retrospective, single-institution, comparative observational study conducted at the Department of Radiation Oncology of Sri Ramachandra Institute of Higher Education and Research, Chennai, India, between January 2022 and January 2024. The inclusion criteria for our study were patients with age ≥18 years, histologically proven prostatic adenocarcinoma, WHO performance status 0-2, high-risk or unfavourable intermediate risk prostate cancer as per National Comprehensive Cancer Network (NCCN) risk stratification [10], and patients who underwent definitive-intent radiotherapy with either moderate hypofractionation or conventional fractionation with concurrent or adjuvant androgen deprivation therapy (ADT) administered according to risk group. The exclusion criteria for this study were patients with evidence of distant metastasis at diagnosis, prior pelvic radiotherapy or radical prostatectomy, and patients with poor performance status. The minimum follow-up duration is 24 months.

**Figure 1:**
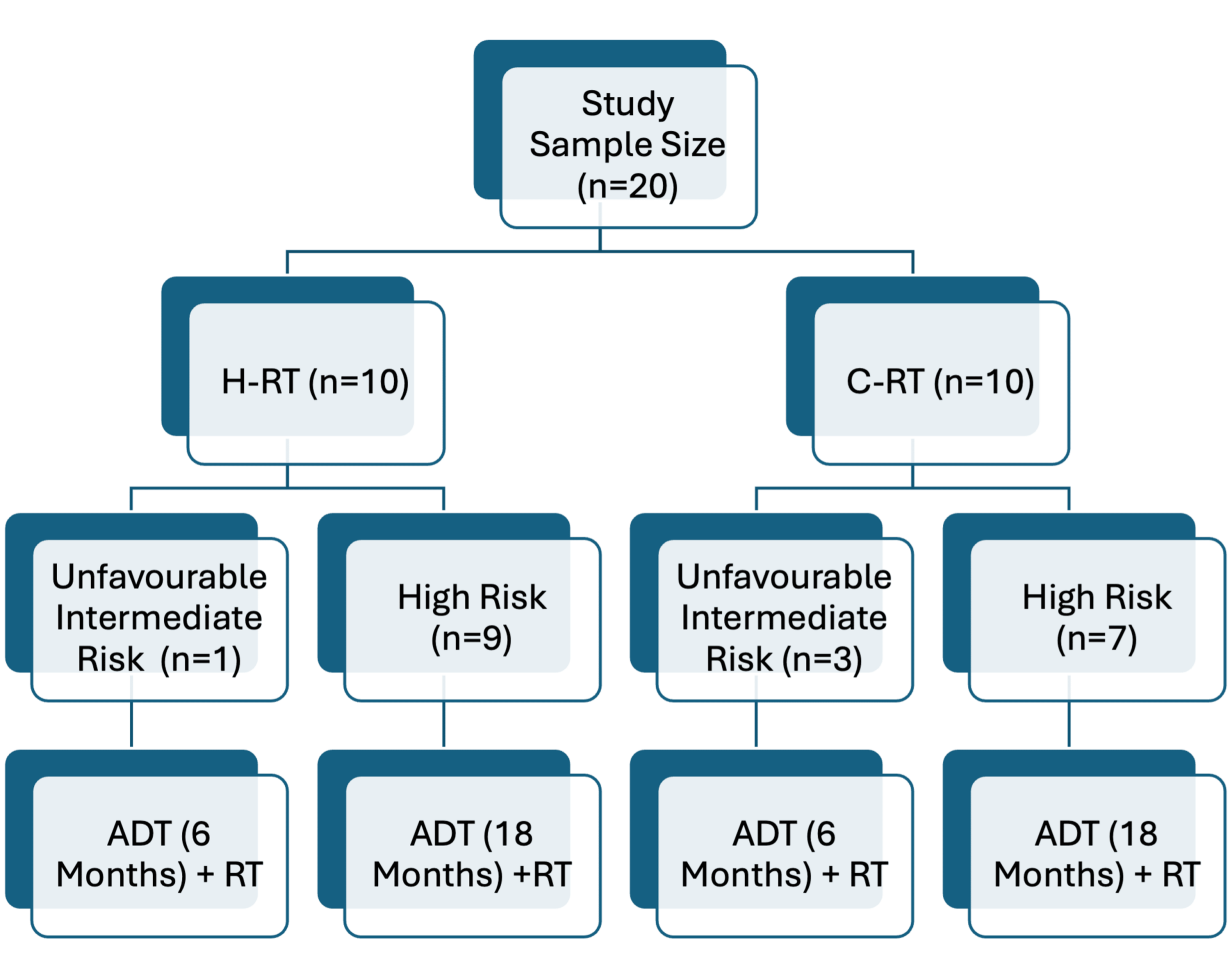
Allocation of the treatment cohorts based on the fractionation schedule H-RT: hypofractionation radiotherapy; C-RT: conventional fractionation radiotherapy; ADT: androgen deprivation therapy; RT: radiotherapy; number of patients: n

Of the 23 patients reviewed, 20 met the inclusion criteria and were included in the final analysis. They were allocated into two treatment cohorts based on the fractionation schedule received, as shown in Figure 1. The two study arms were the patients who received moderate hypo-RT with 70.70 Gy/28 fractions (2.5 Gy/fraction) (n=10), and the comparison group had received conventional fractionated radiotherapy (C-RT) with 80 Gy/40 fractions (2 Gy/fraction) (n=10).

Risk grouping was performed based on the NCCN [10] guidelines, incorporating Gleason score, clinical T stage, and baseline prostate-specific antigen (PSA) level, and was categorized as high-risk or intermediate-risk. The high-risk group consisted of patients with cT3-T4, Grade Group 4 or 5, and a serum PSA value of more than 20 ng/mL, and the unfavorable intermediate-risk group consisted of patients with Grade Group 3, >50% biopsy cores, and 2 or 3 intermediate-risk features.

Patients had received ADT according to disease risk stratification. Patients under unfavorable intermediate-risk prostate carcinoma had received short-term ADT for a duration of six months [11], while those with high-risk disease received long-term ADT for a duration of two years [12]. ADT was administered using luteinizing hormone-releasing hormone (LHRH) agonists or antagonists, based on clinical indication and patient comorbidities. In patients initiated on LHRH agonists, short-term nonsteroidal anti-androgen therapy (bicalutamide) was used to prevent testosterone flare for a period of three weeks. LHRH antagonists were used selectively in patients where rapid testosterone suppression or avoidance of flare was clinically indicated. ADT was initiated in a neoadjuvant setting before radiotherapy and continued concurrently with radiotherapy in all patients, with the total duration individualized according to risk group and the treating physician's discretion.

Radiotherapy technique

The patient was immobilized in the supine position using a pelvic thermoplastic mould, and subsequently, a planning CT scan was done. A bladder-filling protocol was followed, with the patient instructed to empty the bladder and then consume 200 ml of water 20 minutes prior to CT simulation and before each treatment session. A rectal protocol was also maintained using laxatives and a low-fat diet. The CT scan was done with a slice thickness of 3-5 mm from the level of L1 to the mid-thigh. Volume delineation was performed according to the European Society for Radiotherapy and Oncology (ESTRO) guidelines [13]. The gross tumor volume (GTV) for the prostate was defined as the radiologically visible tumor. The clinical target volume (CTV) included the entire prostate gland along with the seminal vesicles. The CTV was expanded by 6 mm in all directions to obtain the prostate planning target volume (PTV), whereas only a 5 mm margin is given posteriorly to spare the rectum. The nodal CTV encompassed the external iliac, internal iliac, obturator, common iliac, and presacral nodal regions, with a 3 mm margin applied to generate the corresponding PTV for nodal volumes.

As per our institutional protocol, all patients with unfavorable intermediate and high risk received treatment to the regional nodes (whole pelvis) and prostate with seminal vesicles. For the treatment arm, we delivered 70 Gy in 28 fractions to the prostate and 50.4 Gy in 28 fractions to the whole pelvis using simultaneous integrated boost (SIB), as shown in Table 1. We generated volumetric modulated arc therapy (VMAT) treatment plans with 6 MV photons that were optimized for conformity and homogeneity. Treatment verification was performed with daily cone beam CT (CBCT) imaging to ensure setup accuracy within 3 mm.

**Table 1:** DOSE PRESCRIPTION H-RT: hypofractionation radiotherapy; C-RT: conventional fractionation radiotherapy

TARGET VOLUME	C-RT		H-RT	
	Total dose (Gy)	Dose per fraction (Gy)	Total dose (Gy)	Dose per fraction (Gy)
Whole pelvis (Elective nodes)	50 Gy	2 Gy	50.4 Gy	1.8 Gy
Prostate + Seminal vesicles	20 Gy	2 Gy	70 Gy	2.5 Gy
Prostate (Boost volume)	10 Gy	2 Gy		
Total dose	80Gy/40 Fractions		70 Gy/28 Fractions	

Patients were followed up weekly during radiotherapy and, following its completion, were reviewed at one month, six months, 12 months, and 24 months. The follow-up assessment included clinical evaluation, PSA measurement, toxicity assessment, and assessment using the International Prostate Symptom Score (IPSS) questionnaire [14].

Objectives

The primary endpoint was to assess the biochemical relapse-free survival (bRFS) at 24 months. Biochemical relapse was defined as per the Phoenix definition, which states that a rise in PSA of ≥2 ng/ml above the nadir [15] value achieved after radiotherapy. A PSA nadir is the lowest level of PSA reached following treatment. The secondary endpoint was to assess the genitourinary symptoms using the IPSS. The IPSS, developed in 1992, is a validated questionnaire developed by the American Urological Association and is used with acknowledgement. It consists of an eight-item self-administered questionnaire that measures the severity of lower urinary tract symptoms (LUTS) and determines the quality of life (QoL) [14]. 

Acute and late gastrointestinal (GI) and genitourinary (GU) toxicities were graded using the RTOG criteria [16]. Acute toxicity was defined as the toxicity that occurs during treatment and leading up to one month post radiotherapy, and sub-acute toxicity was defined as more than one month and less than six months. Late toxicity was considered as adverse effects developing more than six months following treatment. 

The data were retrospectively obtained from patients’ treatment charts who had undergone the standard treatment protocol after obtaining necessary written informed consent. The post-treatment follow-up QoL assessment using IPSS was collected prospectively. An institutional ethics committee approval was obtained for our study (approval number: NI/26/03/117/59).

Statistical analysis

Statistical analysis of the baseline demographic and disease characteristics was summarized using descriptive statistics. Continuous variables were exposed as means with ranges or standard deviations, while categorical variables were reported as frequencies and percentages. Comparisons between the hypo-RT and C-RT groups were performed using an appropriate non-parametric test (chi-square test), owing to the small cohort size and non-normal distribution of data.

The bRFS at 24 months was calculated descriptively for each treatment arm. The incidence and severity of toxicities between treatment arms were compared using Fisher's exact test, given the low event rates and small sample size.

IPSS and IPSS-based QoL scores were summarized using mean values at predefined time points. Changes in IPSS and QoL over time were evaluated descriptively, and intergroup comparisons at each follow-up time point were performed using non-parametric tests, wherever applicable. Given the identical trends observed between treatment arms and the exploratory nature of the analysis, emphasis was placed on clinical patterns rather than formal hypothesis testing.

All statistical tests were two-sided, and a p-value <0.05 was considered statistically significant. However, due to the small sample size and low number of outcome events, statistical findings were interpreted cautiously, with a focus on clinical relevance and consistency of trends rather than statistical significance alone.

## Results

Patient characteristics

A total of 20 patients were included in the study, and patient characteristics were balanced between the two arms, as shown in Table 2. The predominant study population is high-risk prostate cancer patients (90%) in the study group. A statistical comparison of baseline PSA values is shown in Table 3.

**Table 2:** Patient characteristics H-RT: hypofractionation radiotherapy; C-RT: conventional fractionation radiotherapy; PSA: prostate specific antigen

CHARACTERISTICS	NUMBER OF PATIENTS (%) n=20	
	C-RT	H-RT
Age		
60-70 years	5 (50%)	4 (40%)
71-80 years	5 (50%)	6 (60%)
Risk stratification		
Intermediate (Unfavorable)	3 (30%)	1 (10%)
High risk	7 (70%)	9 (90%)
Clinical stage		
T2	6 (60%)	5 (50%)
T3	2 (20%)	3 (30%)
T4	2 (20%)	2 (20%)
N1	6 (60%)	4 (40%)
Baseline PSA		
<10	3 (30%)	1 (10%)
10-20	0 (0%)	0 (0%)
>20	7 (70%)	9 (90%)

**Table 3:** Mean PSA values H-RT: hypofractionation radiotherapy; C-RT: conventional fractionation radiotherapy; PSA: prostate specific antigen

	HYPO-RT	C-RT
PSA mean ± SD (p-value- 0.904)	36.34 ± 24.85	37.58 ± 20.21

**Figure 2:**
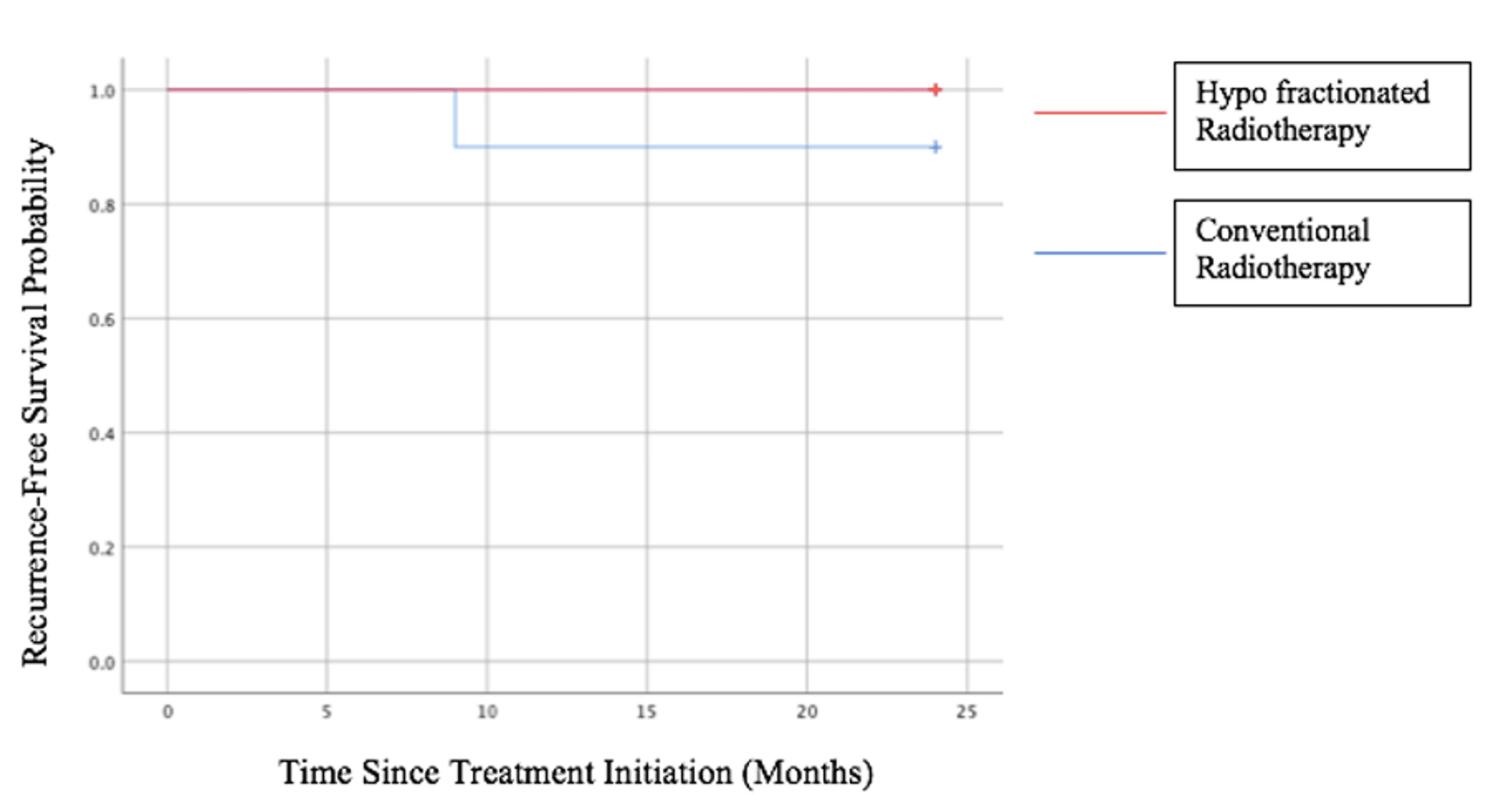
Twenty-four-month biochemical relapse-free survival

The median follow-up period in our study was 24 months, and one patient (10%) in the C-RT arm experienced biochemical relapse. No biochemical failures were recorded in the hypo-RT arm during the study period. The 24-month bRFS was 10 (100%) in the hypo-RT group and nine (90%) in the C-RT group, as shown in Figure 2.

During the follow-up period, one recurrence (10%) occurred in the C-RT group at nine months, whereas no recurrences were observed in the hypo-RT group. The remaining patients in both groups were censored at their last follow-up. Given the very small number of events (n = 1), no meaningful statistical comparison between groups can be established, and the Kaplan-Meier curves show no statistically significant difference (log-rank test not significant).

We had assessed the acute GI and GU toxicities during radiotherapy and up to one month following treatment completion. In both treatment arms, most of the patients experienced Grades 0-1 toxicity (80%), with the common symptoms encountered being increased frequency. Grade 2 acute GI and GU toxicities were observed in a limited number of patients in both groups (20% vs 30%). Notably, no Grade 3 or higher acute GI or GU toxicities were recorded in either treatment arm. The overall incidence and severity of acute toxicity were comparable between hypo-fractionated and conventional radiotherapy, with no statistically significant differences observed, as shown in Table 4.

**Table 4:** Acute GI/GU toxicity H-RT: hypofractionation radiotherapy; C-RT: conventional fractionation radiotherapy; GI: gastrointestinal; GU: genitourinary

STUDY ARM	TOXICITY	DURING TREATMENT		ONE MONTH POST TREATMENT	
		Grades 0-1	Grade 2	Grades 0-1	Grade 2
H-RT (n=10)	GI	8 (80%)	2 (20%)	8 (80%)	2 (20%)
	GU	8 (80%)	2 (20%)	8 (80%)	2 (20%)
C-RT (n=10)	GI	8 (80%)	2 (20%)	7 (70%)	3 (30%)
	GU	7 (70%)	3 (30%)	7 (70%)	3 (30%)

We evaluated late GI and GU toxicities at six, 12, and 24 months following completion of radiotherapy. Late toxicities were predominantly Grades 0-1 in both treatment arms (80% vs 90%), and the commonly encountered symptom was nocturia. Occasional late grade 2 GI or GU toxicities were seen, with similar frequency in the hypo-fractionated and conventional radiotherapy groups. No Grade 3 late toxicities were reported at any follow-up time point in either group. At 24 months, the incidence of GU and GI toxicity remained minimal and did not differ significantly between the treatment arms, as shown in Table 5.

**Table 5:** Late GI/GU toxicity H-RT: hypofractionation radiotherapy; C-RT: conventional fractionation radiotherapy; GI: gastrointestinal; GU: genitourinary

STUDY ARM	TOXICITY	SIX MONTHS POST TREATMENT		12 MONTHS POST TREATMENT		24 MONTHS POST TREATMENT	
		Grades 0-1	Grade 2	Grades 0-1	Grade 2	Grades 0-1	Grade 2
H-RT (n-10)	GI	8 (80%)	2 (20%)	100%	0 (0%)	10 (100%)	0 (0%)
	GU	9 (90%)	1 (10%)	9 (90%)	1 (10%)	10 (100%)	0 (0%)
C-RT (n-10)	GI	10 (100%)	0 (0%)	10 (100%)	0 (0%)	10 (100%)	0 (0%)
	GU	9 (90%)	1 (10%)	10 (100%)	0 (0%)	10 (100%)	0 (0%)

IPSS was assessed at baseline, during radiotherapy, and at one, six, 12, and 24 months post treatment. Based on IPSS, the symptoms were graded as mild, moderate, and severe based on the scores obtained [13].

Both treatment groups demonstrated a transient increase in IPSS scores during radiotherapy, indicative of acute urinary symptoms. Subsequently, IPSS scores improved over the follow-up period, approaching baseline values by 6 to 12 months. 

At 24 months, no clinically meaningful difference in IPSS scores was observed between the hypo-RT and conventional RT groups (moderate symptoms 20% vs 30%) as shown in Table 6.

**Table 6:** Urinary symptoms based on IPSS RT: radiotherapy; H-RT: hypofractionation radiotherapy; C-RT: conventional fractionation radiotherapy; IPSS: International Prostate Symptom Score

		BASELINE	DURING RT	ONE MONTH	SIX MONTHS	12 MONTHS	24 MONTHS
H-RT	Mild	5 (50%)	2 (20%)	1 (10%)	3 (30%)	7 (70%)	8 (80%)
	Moderate	5 (50%)	8 (80%)	9 (90%)	7 (70%)	2 (20%)	2 (20%)
C-RT	Mild	6 (60%)	3 (30%)	1 (10%)	3 (30%)	7 (70%)	7 (70%)
	Moderate	4 (40%)	7 (70%)	9 (90%)	7 (70%)	3 (30%)	3 (30%)

QoL assessment 

At baseline, the mean QoL score was 1.8 in both the hypo-RT and C-RT groups, indicating comparable pre-treatment urinary-related QoL. A transient deterioration in QoL was observed at one month following radiotherapy in both cohorts, with the mean QoL score increasing to 2.8 in each group, consistent with acute treatment-related urinary symptoms.

Subsequently, QoL scores improved progressively in both treatment arms. At six months post treatment, the mean QoL score improved to 1.7 in both groups. At 12 and 24 months, QoL scores remained stable, with a mean of 1.1 in both the hypo-RT and C-RT cohorts. Overall, no clinically meaningful difference in mean QoL scores was observed between the two fractionated schedules at any assessed time point. 

## Discussion

The present retrospective study evaluated biochemical control and toxicity outcomes in patients with intermediate- and high-risk prostate cancer treated with definitive external beam radiotherapy using either moderate hypofractionation or conventional fractionation, in combination with risk-adapted ADT. Despite the small cohort size, the study provides clinically relevant real-world data supporting the feasibility and safety of hypo-RT using the schedule of 70Gy in 28 fractions (SIB) in routine practice. Indian data supporting the use of this fractionation schedule is sparse.

In our study, at a median follow-up of 24 months, excellent biochemical control was observed across both treatment arms. Notably, no biochemical relapses were documented in the hypo-RT cohort, whereas a single biochemical relapse occurred in the conventional fractionation arm. Although the difference in bRFS was not statistically significant due to the limited number of events, these findings are consistent with the growing body of evidence demonstrating at least equivalent oncological outcomes with hypo-fractionated schedules. Importantly, the observed biochemical outcomes should be interpreted cautiously, given the short follow-up and the exploratory nature of the analysis.

The results of this study closely align with those of the landmark CHHiP trial [2], demonstrating non-inferiority of moderate hypofractionation (60 Gy in 20 fractions or 57 Gy in 19 fractions) compared with conventional fractionation (74 Gy in 37 fractions), with a reported five-year biochemical failure-free survival of 90.6% and 85.9% vs. 88.3% [2]. In our cohort, the 24-month bRFS was 100% in the hypo-fractionated arm and 90% in the conventional arm, consistent with the disease control observed in the CHHiP trial [2], despite a higher proportion of high-risk, node-positive patients in our population. These findings suggest that modern hypofractionation delivers comparable early biochemical control in real-world practice to that observed in large international randomized trials.

In contrast to the CHHiP trial [2], our study included treating the elective lymph nodes as well as the primary. Despite the increase in the radiation volume, our toxicity outcomes in the present analysis were reassuring. Acute GI and GU toxicities were predominantly mild, with most patients experiencing Grades 0-1 events. Importantly, no Grade ≥3 acute toxicity was observed in either treatment arm. Late toxicity outcomes were also favorable, with low rates of Grade 2 GI and GU toxicity (10%), and a complete absence of Grade ≥3 acute toxicity was observed in either treatment arm. This toxicity profile mirrors the CHHiP trial [2], in which Grade ≥2 late GI toxicity was 11.9% and 11.3% vs. 13.7%, and Grade ≥2 GU toxicity was 11.7% and 6.6% vs. 9.1%, with very low rates of Grade ≥3 events, confirming that hypofractionation does not lead to excess late morbidity when delivered with modern techniques. The comparable toxicity observed in our study further supports the radiobiological premise that prostate cancer, with its low α/β, benefits from larger fraction sizes without a proportional increase in late normal tissue toxicity [17-21].

Among the Indian data, a study by Mallick et al. [9] demonstrated the use of hypo-RT in high-risk prostate cancer, and the treatment volumes included elective nodal irradiation and prostate, with only 15% with Grade 2 GI toxicity and 19.2% with Grade 2 GU toxicity. The late toxicity observed in our study is comparable with 10% Grade 2 GU and 20% Grade 2 GI toxicity. Another study by Soni et al. [8] had similar rates of Grade 2 GI and GU toxicities (15.5% & 11.9%).

Urinary symptom burden, as assessed by the IPSS, worsened transiently during radiotherapy in both groups, followed by gradual improvement during follow-up. By 24 months, IPSS values had largely returned towards baseline levels, with no clinically meaningful difference between treatment arms. This pattern closely reflects the CHHiP QoL data, where acute urinary symptoms peaked during treatment but resolved over time without long-term detriment [2]. 

In our study, all high-risk patients had completed two years of ADT with two cycles as neoadjuvant and continued post radiotherapy for two years, and the unfavorable intermediate risk had two doses of ADT prior to RT. The use of ADT in all patients reflects the contemporary standard of care for intermediate- and high-risk prostate cancer. By ensuring risk-adapted and uniform use of ADT across both fractionation cohorts, potential confounding related to systemic therapy was minimized, allowing a more accurate comparison of radiotherapy fractionation schedules. The absence of excess toxicity despite combined-modality treatment further underscores the tolerability of hypo-fractionated regimens when delivered with modern image-guided techniques.

The main drawbacks of this study are its limited sample size and follow-up duration. The low number of biochemical failure events precluded robust statistical comparison, and the findings should therefore be interpreted as hypothesis-generating rather than definitive. Nevertheless, the strength of this analysis lies in its consistent treatment protocols, detailed toxicity assessment, and real-world data, which are particularly relevant in high-volume centers within resource-constrained settings. Notably, the close concordance of biochemical control, toxicity, and QoL outcomes with those reported in the CHHiP trial [2] supports the external validity of our findings. These findings indicate that moderate hypo-RT did not adversely impact long-term urinary-based QOL when compared against the patients who underwent C-RT.

## Conclusions

Moderate hypo-RT takes advantage of the radiobiological properties of prostate cancer with its low α/β ratio in achieving comparable oncologic outcomes with minimal early and late toxicities using high-precision radiotherapy techniques. Hence, moderate hypo-RT with reduced overall treatment time in comparison to conventional fractionation supports its use as a standard treatment approach in the management of prostate cancer.
